# Variation of heart and lung radiation doses according to setup uncertainty in left breast cancer

**DOI:** 10.1186/s13014-021-01806-5

**Published:** 2021-04-20

**Authors:** Sunmin Park, Chai Hong Rim, Won Sup Yoon

**Affiliations:** Department of Radiation Oncology, Ansan Hospital, Korea University, 123 Jeokgeum-ro, Danwon-gu, Ansan, Gyeonggi-do 15355 Republic of Korea

**Keywords:** Breast cancer, Radiotherapy, Set-up uncertainty, Deep inspiration breath holding, Heart

## Abstract

**Purpose:**

Breast radiotherapy set-up is often uncertain. Actual dose distribution to normal tissues could be different from planned dose distribution. The objective of this study was to investigate such difference in dose distribution according to the extent of set-up error in breast radiotherapy.

**Materials and methods:**

A total of 50 Gy with fraction size of 2 Gy was given to 30 left breasts with different set-ups applying a deep inspiration breath holding (DIBH) or a free breathing (FB) technique. Under the assumption that errors might come from translational axes of deep or caudal directions, the isocenter was shifted from the original tangential alignment every 2.5 mm to simulate uncertainty of deep and caudal tangential set-up in DIBH and FB. Changes were evaluated for dosimetric parameters for the heart, the left ventricle (LV), the left anterior descending coronary artery (LAD), and the ipsilateral lung.

**Results:**

On the original plan, mean doses of heart and ipsilateral lung were 2.0 ± 1.1 Gy and 3.7 ± 1.4 Gy in DIBH and 8.4 ± 1.3 Gy and 7.8 ± 1.5 Gy in FB, respectively. The change of dose distribution for the heart in DIBH was milder than that in FB. The deeper the tangential set-up, the worse the heart, LV, LAD, and ipsilateral lung doses, showing as much as 49.4%, 56.4%, 90.3%, and 26.1% shifts, respectively, in 5 mm DIBH setup. The caudal set-up did not show significant dose difference. In multiple comparison of DIBH, differences of mean dose occurred in all 7.5 mm deep set-ups for the heart (*p* = 0.025), the LV (*p* = 0.049), and LAD (*p* = 0.025) in DIBH.

**Conclusions:**

To correct set-up error over indicated limitation for deep tangential set-up in DIBH at 5 mm action level, mean heart and ipsilateral lung doses are expected to increase approximately 50% and 25%, respectively.

## Introduction

The issue of cardiac toxicity after breast radiotherapy was raised in the early 2000’s. It has the following features. First, atherosclerotic change can cause coronary damage [1]. Second, cardiac events have continuously increased over a decade after radiotherapy. Therefore, long-term observation is needed [2, 3]. Third, pre-existing risk factors such as smoking, old age, obesity, cardio-metabolic risk factors of hypertension and diabetes, and other cardiovascular or cerebrovascular disease can affect cardiac toxicity [4–6]. Most importantly, cardiac toxicity increases gradually per mean heart dose without a clear threshold [7]. Therefore, radiation dose for the heart should be avoided as low as reasonably achievable. The expert consensus has recommended deep inspiration breath hold (DIBH), prone position, and/or heart blocks to minimize heart dose [8]. The technique of DIBH is currently being applied to left breast cancer in many institutions. One study has compared DIBH and free breathing (FB) and found that DIBH can decrease 29.2% of mean heart dose and 43.5% of mean left anterior descending coronary artery (LAD) dose [9]. In a Asian cohort, the mean heart dose reduction throughout DIBH compared to FB is 47%. This effect is more significant in those with low body mass index [10].

Tangential irradiation method is considered the most common and effective method in whole breast radiotherapy to minimize radiation dose to the opposite breast. In recent years, field-in-field techniques have been combined to further reduce ambient dose. However, because of uneven body surface, irregular breathing, incomplete body fixation, soft breast tissue, and so on, set-up uncertainty has become a limiting factor for distributing radiation dose as planned [11]. Then, with a deep set-up which is harmful to normal tissues such as the heart or the ipsilateral lung, to what extent is the radiation dose exceeded and what is the acceptable action level to correct set-up errors in clinical practice? No studies have addressed these questions. Thus, the objective of this study was to investigate dose distribution in the organs at risk (OARs) of heart, sub-segments of heart, and ipsilateral lungs according to set-up uncertainty, analyze characteristics, and assume the dose increase of OARs according to the action level of set-up error.

## Methods and materials

### Patients

Of patients receiving breast conserving surgery including sentinel lymph node biopsy with clinical T1-2N0 stage, those with left breast cancer who underwent adjuvant radiotherapy for whole breast alone were reviewed. Neoadjuvant chemotherapy was allowed and pathological N1 stage not to need additional axillary field was included. Each of 15 patients were identified in DIBH and FB. Because our institution has applied DIBH since October 2019, 15 patients consecutive from November 2019 to February 2020 were selected for the DIBH group. To minimize the effect of OARs by the different characteristics of body contour between DIBH and FB groups, FB group was selected based on clinical target volume (CTV) of breast [12]. Of 33 patients from October 2017 to May 2018, 15 patients for the FB group were paired with the DIBH group considering the approximate CTV. This retrospective study was approved by the Institutional Review Board of Ansan Hospital, Korea University, Republic of Korea.

For computed tomography (CT) simulation, a Brilliance Big Bore Oncology CT system (Philips Medical Systems, Nederland) and a Breastboard (Civco, Orange City, IA, USA) as immobilization devices were utilized. All set-ups were done at a supine position with an elevation of both arms above the head. CT contrast was administrated to enhance vascular structures and tumor bed. CT scans were sliced with a thickness of 3 mm. While there was no education of breathing control for the FB group, the concept of DIBH was explained to patients on the first consultation day. Self-training was proceeded to hold their breath for a minimum of 20 s with a feeling of inhaling a small 1000 cc plastic bottle for the DIBH group. Patients who had difficulty holding their breath for more than 20 s in the prior practice were excluded in DIBH. Our institution performed daily verification using the electronic portal images, Portal Vision aS1000 (Varian Medical System, Palo Alto, CA, USA) and checked the stability of chest wall during DIBH using the Real Time Position Management system (Varian Medical System).

### Radiotherapy planning

Dose distribution was calculated with a radiation therapy planning system, a Varian Eclipse version 15.1 (Varian Medical System) using Anisotropic Analytical Algorithm. For CTV of the whole breast, ESTRO guideline was considered and 5 mm from the body surface of the CTV (4 mm for the small sized breast less than 400 cc) was edited [13]. To delineate OARs of the heart and sub-segments (left ventricle (LV) and LAD) of heart, the report of cardiac contouring atlas by Duane et al. [14] was used as a reference. The ipsilateral lung was delineated with CT window level and width of 0/1000 HU. The prescribed dose was modified as 50 Gy with 25 fractions to all patients to cover CTV > 95% with prescribed dose > 95% without maximum CTV dose > 107%. The field-in-field technique using 6MV photon beams was made. For this study, delineation of the CTV and OARs and treatment plans were newly verified in consultation with two experienced radiation oncologists (Yoon and Rim).

### Study simulation

**Fig. 1:**
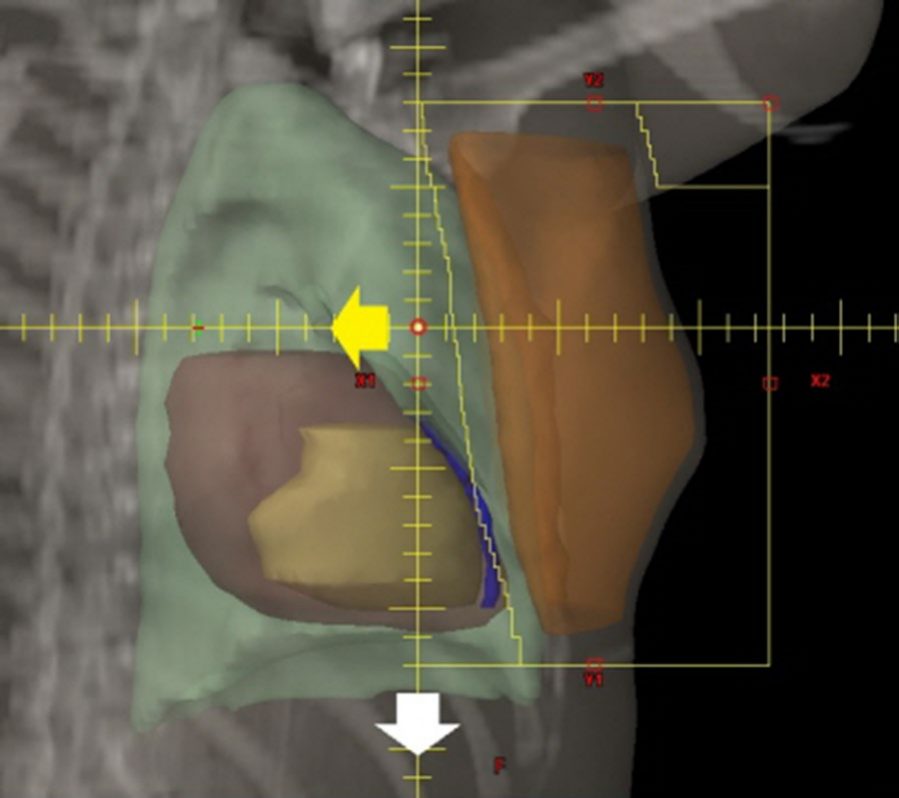
Beam’s eye view of medial tangential field. After the organ at risks and target volume were drawn (Heart; brown, Left ventricle; yellow, left anterior descending coronary artery; blue, Lung; green, breast target volume; Orange) and original tangential fields were aligned, translation movement of deep (yellow arrow) and caudal (white arrow) directions was simulated in every 2.5 mm and 5 mm interval till 15 mm set-up error, respectively

For this study, we simulated two main conditions of set-up error in a separate way: (1) in the deep direction (virtual perpendicular direction from the original tangential alignments); and (2) in the caudal direction of iso-center. Under the assumption that errors from rotational position and other directions were corrected, the isocenter shifted in deep and caudal directions every 2.5 mm until reaching 15 mm error (Fig. 1). After the isocenter was moved as much as each set-up error, dose distribution was recalculated. Thus, uncertainties of deep tangential and caudal set-up were simulated.

### Statistics

Mean dose, V10Gy, and V20Gy of heart, mean dose and V20Gy of LV, mean dose and V30Gy of LAD, and mean dose, V10Gy, and V30Gy of ipsilateral lung were measured. Each parameter was presented with a mean (M) ± standard deviation (SD). The difference between FB and DIBH groups was examined with an independent two sample *T* test. The difference in dose distribution between the original plan and each simulated set-up plan was calculated in both absolute dose (Gy) and the relative ratio on the basis of the original dose (%). Then, it was compared with a paired T-test. In addition, multiple comparisons were performed with LSD (least significant difference) method to compare differences between simulated set-ups and to search the point as action level. A two-sided *p* < 0.05 was considered significant. SPSS 20.0 (IBM SPSS Inc., Chicago, IL, USA) was used for all statistical analyses.

## Results

### Patient characteristics

After matching DIBH and FB groups according to CTV, 15 patients in each group were selected. Median age was 54 years (range, 41–66 years) for the DIBH group and 48 years (range, 39–63 years) for the FB group. For OARs, the ipsilateral lung (mean 923.1 ml vs. 1600.7 ml, *p* < 0.001) was larger in DIBH group. However, there was no volume difference of heart or its sub-segments (Table 1).

**Table 1:** Patient characteristics

	Deep inspiration breath hold (N = 15)		Free breath (N = 15)		
pT-stage 0: 1: 2	1^a^: 9: 5		0: 9: 6		
pN-stage 0: 1	14: 1		14: 1		

	Median	Range	Median	Range	*P* value^b^
Age (years)	54	41–66	48	39–63	0.715
Tangential angle (°)	311	306–318	312	302–316	1.000
Field height (cm)	20	17–22	20	18–21.5	0.245

	Mean	SD	Median	SD	*P* value^c^
Clinical target volume (ml)	682.2	280.6	669.2	242.9	0.892
Heart (ml)	616.0	82.2	645.1	81.5	0.339
Left ventricle (ml)	151.1	22.6	160.7	26.8	0.298
Left anterior descending coronary artery (ml)	1.1	0.1	1.1	0.1	0.271
Lung, ipsilateral (ml)	1600.7	409.4	923.1	227.6	< 0.001

^a^Patient had complete response after receiving neoadjuvant chemotherapy

^b^The median value was compared through a Mann–Whitney U-test

^c^The mean value was compared through an independent two sample *T* test

### Original plan of DIBH and FB

On the original plan, mean doses of the heart were 2.0 ± 1.1 Gy (range, 0.85–4.95 Gy) and 3.7 ± 1.4 Gy (range, 1.7–6.15 Gy) in DIBH and FB groups, respectively. Mean doses of LV and LAD were 3.3 ± 2.5 Gy and 17.8 ± 12.7 Gy in DIBH and 6.0 ± 2.3 Gy and 35.9 ± 9.6 Gy in FB, respectively. These results showed benefits of DIBH for decreasing doses for heart and its sub-segments in comparison with FB. Mean doses of the ipsilateral lung were 8.4 ± 1.3 Gy (range, 6.1–11.1 Gy) and 7.8 ± 1.5 Gy (range, 5.7–11.4 Gy) in DIBH and FB groups, respectively.

### Extent of dose difference

The deeper the tangential set-up was, the worse the mean heart and ipsilateral lung dose became as much as 0.23 Gy and 0.44 Gy per mm to 10 mm shift in DIBH, and 0.37 Gy and 0.46 Gy in FB, respectively (all *p* < 0.001). However, caudal set-up did not affect dose distribution of OARs in DIBH or FB group (Table 2).

**Table 2:** Dose distribution for the organ at risks in terms of deep and caudal set-up errors in deep inspiration breath hold and free breath (Mean ± (Standard deviation))

	Heart			Left ventricle		Left anterior descending coronary artery		Lung, ipsilateral		
	Mean (Gy)	V10Gy (%)	V20Gy (%)	Mean (Gy)	V20Gy (%)	Mean (Gy)	V30Gy (%)	Mean (Gy)	V10Gy (%)	V30Gy (%)
Deep inspiration breath hold (N = 15)										
No shift	2.0 (1.1)	2.8 (2.7)	2.1 (2.3)	3.3 (2.5)	3.8 (5.1)	17.8 (12.7)	29.3 (31.2)	8.4 (1.3)	19.7 (3.1)	12.8 (2.7)
Deep										
2.5 mm	2.5 (1.4)	3.8 (3.2)	2.9 (2.8)	4.1 (2.9)	5.4 (6.3)	22.2 (13.2)	38.7 (31.8)	9.4 (1.3)	22.1 (3.2)	15.0 (2.9)
5 mm	3.0 (1.6)	4.9 (3.8)	3.9 (3.3)	5.0 (3.4)	7.4 (7.3)	26.9 (13.0)	50.9 (30.6)	10.5 (1.3)	24.6 (3.2)	17.2 (2.9)
7.5 mm	3.6 (1.8)	6.3 (4.3)	5.1 (3.8)	6.2 (3.9)	9.9 (8.4)	31.4 (12.9)	61.7 (29.3)	11.6 (1.4)	27.1 (3.3)	19.6 (2.9)
10 mm	4.3 (2.1)	7.9 (4.8)	6.6 (4.4)	7.5 (4.3)	12.7 (9.5)	35.1 (13.1)	69.9 (30.5)	12.8 (1.4)	29.6 (3.4)	22.0 (3.0)
12.5 mm	5.0 (2.3)	9.7 (5.3)	8.2 (4.90	8.9 (4.7)	15.8 (10.5)	38.0 (13.1)	76.0 (32.0)	13.9 (1.4)	32.0 (3.4)	24.4 (3.0)
15 mm	5.9 (2.5)	11.7 (5.8)	10.0 (5.4)	10.5 (5.1)	19.3 (4.3)	40.7 (12.1)	81.0 (30.4)	15.0 (1.5)	34.5 (3.5)	26.9 (3.1)
Caudal										
5 mm	2.1 (1.2)	3.0 (2.8)	2.3 (2.4)	3.5 (2.6)	4.1 (5.4)	18.7 (13.0)	32.3 (30.8)	8.6 (1.2)	20.3 (2.9)	13.4 (2.6)
10 mm	2.2 (1.2)	3.2 (3.0)	2.5 (2.5)	3.7 (2.7)	4.5 (5.7)	19.6 (13.2)	32.9 (32.6)	8.9 (1.1)	20.9 (2.7)	14.0 (2.3)
15 mm	2.3 (1.3)	3.5 (3.2)	2.7 (2.7)	3.9 (2.9)	4.9 ()6.1)	20.6 (13.5)	34.7 (32.1)	9.1 (1.0)	21.4 (2.6)	14.5 (2.1)
Free breath (N = 15)										
No shift	3.7 (1.4)	6.6 (3.2)	5.3 (2.8)	6.0 (2.3)	9.1 (5.2)	35.9 (9.6)	72.6 (24.7)	7.8 (1.5)	17.7 (3.6)	11.9 (3.2)
Deep										
2.5 mm	4.5 (1.5)	8.4 (3.5)	7.0 (3.2)	7.4 (2.5)	12.2 (5.7)	40.2 (6.8)	83.8 (15.5)	8.9 (1.5)	20.2 (3.7)	14.2 (3.3)
5 mm	5.4 (1.7)	10.4 (3.9)	8.8 (3.5)	9.0 (2.7)	15.7 (6.1)	42.9 (5.4)	89.0 (12.2)	10.1 (1.6)	22.7 (3.8)	16.7 (3.4)
7.5 mm	6.4 (1.8)	12.7 (4.2)	10.9 (3.9)	10.7 (2.8)	19.5 (6.5)	44.7 (4.3)	92.0 (10.0)	11.2 (1.6)	25.2 (3.8)	19.1 (3.5)
10 mm	7.4 (1.9)	15.1 (4.5)	13.2 (4.2)	12.4 (2.9)	23.5 (6.9)	46.1 (3.5)	94.5 (7.8)	12.4 (1.7)	27.8 (3.9)	21.6 (3.5)
12.5 mm	8.6 (2.1)	17.7 (4.7)	15.7 (4.5)	14.3 (3.0)	27.7 (7.2)	47.2 (2.6)	96.8 (5.8)	13.6 (1.7)	30.4 (4.0)	24.2 (3.6)
15 mm	9.8 (2.2)	20.5 (5.0)	18.3 (4.7)	16.3 (3.1)	32.1 (7.4)	48.0 (1.8)	98.2 (3.9)	14.8 (1.8)	33.0 (4.0)	26.7 (3.7)
Caudal										
5 mm	3.8 (1.5)	6.9 (3.4)	5.6 (3.0)	6.2 (2.5)	9.6 (5.5)	36.2 (10.0)	74.2 (24.3)	7.9 (1.4)	17.9 (3.5)	12.2 (3..1)
10 mm	3.9 (1.6)	7.2 (3.7)	5.9 (3.3)	6.4 (2.6)	10.1 (5.9)	36.4 (10.6)	74.2 (25.6)	8.0 (1.4)	18.1 (3.4)	12.5 (3.0)
15 mm	4.1 (1.7)	7.5 (4.0)	6.2 (3.6)	6.6 (2.8)	10.6 (6.3)	36.5 (11.2)	72.6 (30.3)	8.1 (1.4)	18.3 (3.3)	12.7 (3.0)

The prescribed dose was 50 Gy with 25 fractions in all plans

**Fig. 2:**
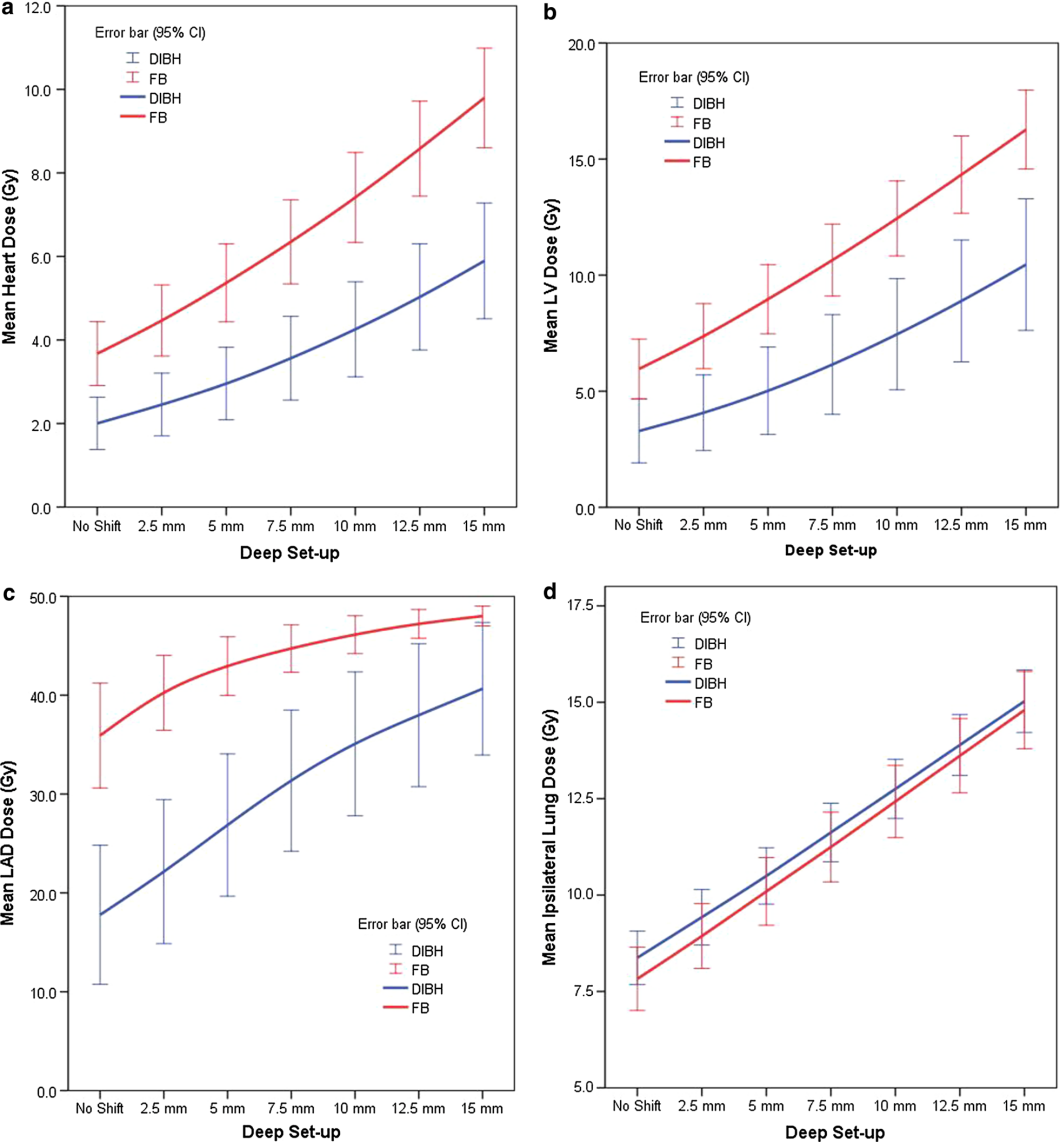
The increase of mean dose distribution (Gy) from the original plan to worsen errors in deep set-up error. **a** Heart, **b** left ventricle, **c** left anterior descending coronary artery, and **d** ipsilateral lung

**Fig. 3:**
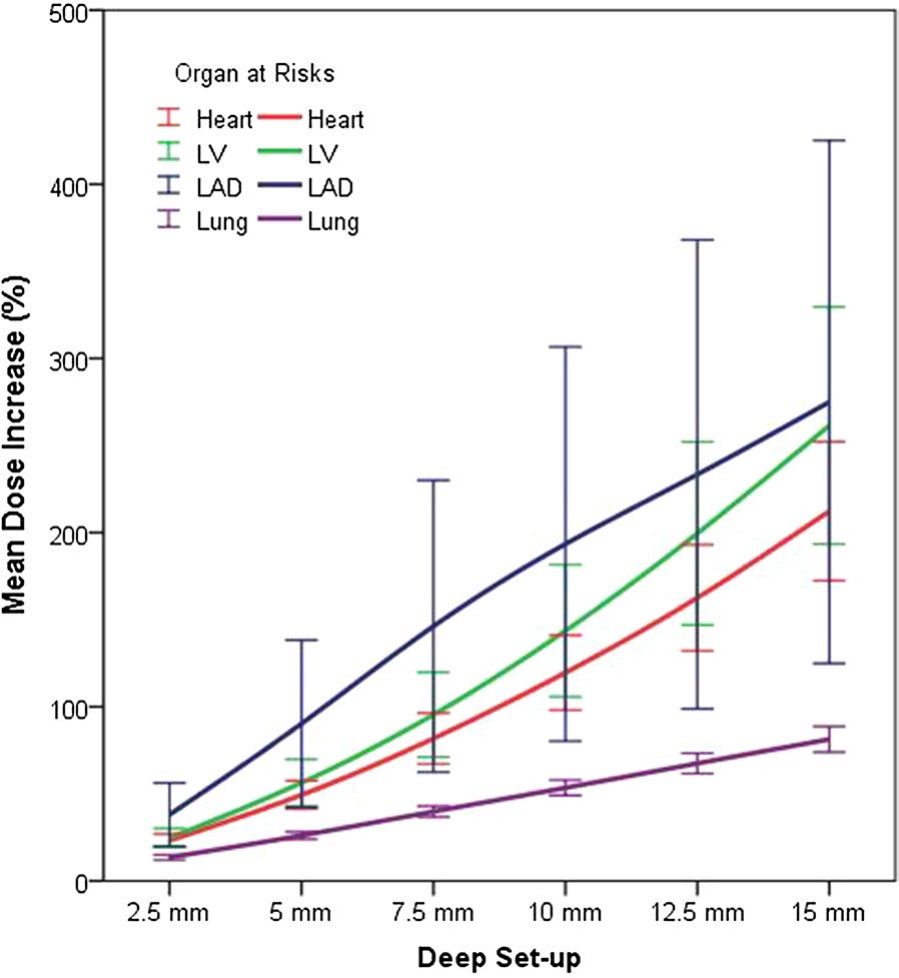
The changes of mean dose distribution (%) of worsen errors against the original plan in deep set-up error of deep inspiration breath hold

Differences between DIBH and FB (∆FB (Deep set-up – Original plan) – ∆DIBH (Deep set-up – Original plan)) of mean heart and LV doses were 0.73 Gy (95% CI 0.42–1.04 Gy, *p* < 0.001) and 1.27 Gy (95% CI 0.65–1.88 Gy, *p* < 0.001) at 5 mm and 1.49 Gy (95% CI 0.85–2.12 Gy, *p* < 0.001) and 2.31 Gy (95% CI 1.06–3.57 Gy, *p* = 0.001) at 10 mm deeper set-up (Fig. 2a, b). These results suggested that DIBH showed a relatively favorable dose distribution than FB in the case of deeper set-up uncertainty for the heart and the LV. Mean LAD dose with a deep set-up of 10 mm in DIBH was similar with the original plan of FB (Fig. 2c). Mean ipsilateral lung dose showed a qualitative increase of about 2 Gy per 5 mm deeper set-up regardless breath technique. It was 2.15 ± 0.17 Gy versus 2.29 ± 0.25 Gy at 5 mm deep set-up and 4.40 ± 0.35 Gy versus 4.63 ± 0.52 Gy at 10 mm deep set-up. (Fig. 2d) Mean heart and LV doses of DIBH increased 49.4 ± 14.5% and 56.4 ± 24.2% at 5 mm deeper set-up and 119.6 ± 38.9% and 143.6 ± 68.5% at 10 mm deeper set-up, respectively (Fig. 3).

In multiple comparison of DIBH, mean doses were significantly different in all 7.5 mm deep set-ups for the heart (mean difference: 1.56 Gy, *p* = 0.025), the LV (mean difference: 2.87 Gy, *p* = 0.049), and the LAD (mean difference: 13.58 Gy, *p* = 0.025) in DIBH. The mean dose difference was more sensitive in FB with a 5 mm deep set-up for the heart (mean difference: 1.69 Gy, *p* = 0.012), the LV (mean difference: 3.01 Gy, *p* = 0.004), and the LAD (mean difference: 7.02 Gy, *p* = 0.001). For the mean ipsilateral lung dose, the difference was developed at 2.5 mm deep set-up in DIBH (mean difference: 1.05 Gy, *p* = 0.037) and at 5 mm deep set-up in FB (mean difference: 2.27 Gy, *p* < 0.001) (Table 3). If the practical action level to determine re-setup was given as 5 mm deep in DIBH, the maximum increases of V20 Gy for the heart, V20 Gy for the LV, V30 Gy for the LAD, and V30 Gy for ipsilateral lung were expected till 1.8 ± 1.1%, 3.6 ± 2.5%, 21.6 ± 20.3%, and 4.5 ± 0.4%, respectively.

**Table 3:** Mean dose differences of worsen errors against the original plan in deep set-up error

	Heart			Left ventricle			Left anterior descending artery			Lung, Ipsilateral		
	Mean difference	*P* value	95% CI	Mean difference	*P* value	95% CI	Mean difference	*P* value	95% CI	Mean difference	*P* value	95% CI
Deep inspiration breath hold (N = 15)												
2.5 mm	− 0.45	0.512	− 1.81/0.91	− 0.79	0.586	− 3.64/2.07	− 4.37	0.354	− 13.70/4.95	− 1.05	0.037	− 2.04/− 0.07
5 mm	− 0.95	0.167	− 2.31/0.40	− 1.73	0.232	− 4.59 /1.12	− 9.08	0.056	− 18.41/0.24	− 2.12	0.000	− 3.11/− 1.14
7.5 mm	− 1.56	0.025	− 2.92/− 0.20	− 2.87	0.049	− 5.72/− 0.01	− 13.58	0.005	− 22.91/− 4.25	− 3.25	0.000	− 4.24/− 2.26
10 mm	− 2.25	0.001	− 3.61/− 0.90	− 4.17	0.005	− 7.02/− 1.31	− 17.30	0.000	− 26.62/− 7.97	− 4.38	0.000	− 5.37/− 3.39
12.5 mm	− 3.03	0.000	− 4.38/− 1.67	− 5.60	0.000	− 8.46/− 2.75	− 20.20	0.000	− 29.52/− 10.87	− 5.52	0.000	− 6.51/− 4.53
15 mm	− 3.89	0.000	− 5.25/− 2.53	− 7.17	0.000	− 10.02/− 4.31	− 22.87	0.000	− 32.20/− 13.54	− 6.65	0.000	− 7.64/− 5.67
Free breath (N = 15)												
2.5 mm	− 0.79	0.234	− 2.11/0.52	− 1.41	0.167	− 3.42/0.60	− 4.33	0.032	− 8.23/− 0.38	− 1.11	0.067	− 2.30/0.08
5 mm	− 1.69	0.012	− 3.01/− 0.38	− 3.00	0.004	− 5.01/− 0.99	− 7.02	0.001	− 10.97/− 3.07	− 2.27	0.000	− 3.46/− 1.08
7.5 mm	− 2.68	0.000	− 3.99/− 1.36	− 4.69	0.000	− 6.70/− 2.68	− 8.81	0.000	− 12.76/− 4.86	− 3.42	0.000	− 4.61/− 2.23
10 mm	− 3.74	0.000	− 5.01/− 2.43	− 6.48	0.000	− 8.49/− 4.47	− 10.21	0.000	− 14.16/− 6.26	− 4.60	0.000	− 5.79/− 3.41
12.5 mm	− 4.91	0.000	− 6.22/− 3.59	− 8.37	0.000	− 10.38/− 6.36	− 11.29	0.000	− 15.24/− 7.34	− 5.79	0.000	− 6.98/− 4.60
15 mm	− 6.12	0.000	− 7.44/− 4.81	− 10.31	0.000	− 12.32/− 8.30	− 12.08	0.000	− 16.03/− 8.13	− 6.97	0.000	− 8.16/− 5.78

Multiple comparisons with LSD method

## Discussion

This study evaluated effects of set-up errors that might occur in daily practice of tangential breast irradiation on OARs. The concave part of the heart was included in the tangential radiation field first. As deep set-up error increases, dose change steeply increases in a certain range. On the other hand, since lungs are originally planned with the concave part already included in the tangential field, a quantitative dose increase occurs according to deep set-up error per mm. Based on our calculations, it seems possible that unexpected cardiac and pulmonary toxicity on original radiotherapy planning could be presented due to an inadvertent delivery without adjusting deep set-up error.

For heart and its sub-segments, DIBH was insensitive to deterioration of mean dose for the same set-up error compared to FB. It was more beneficial given that the original planned dose in DIBH was smaller than FB for those OARs. Based on a previous study showing that the relative risk of acute major coronary events is 7.4% per Gy [7], DIBH can reduce major coronary events by roughly 5% for the same 5 mm deep set-up compared to FB by reducing 0.7 Gy of mean heart dose. Although results with DIBH were evaluated to be somewhat worse for the ipsilateral lung than those with FB, the difference was manageable considering the threshold dose of pulmonary toxicity.

It is known that each radiotherapy facility in Korea accounts for 25% of the burden of breast radiotherapy [15]. Of course, it would be ideal if all set-up errors can be corrected and the planned dose can be presented. However, there is a necessity about an action level as long as there are practical limitations. When we only examined statistical changes in mean value of heart dose for DIBH, not the risk of cardiac toxicity, the significant differences began to be shown from 7.5 mm deep set-up. Therefore, we carefully assumed an action level of 5 mm deep in DIBH. The obvious one is that the degree of action level in FB needed to be strictly set. When this pattern of deep set-up error within 2.5 mm and 5 mm consistently developed in the entire radiotherapy period of FB, our study showed that approximately 23% and 49% of heart dose could increase, respectively, in comparison with the original plan. In modern series, median mean heart doses for left side breast cancer applying conventional (50 Gy in 25 fractions, DIBH 27.8%) and hypofraction (42.6 Gy in 16 fractions, DIBH 14.6%) schedule were 2.16 Gy and 1.47 Gy, respectively [16]. When our study results of DIBH are applied to the above study, cardiac dose can rise up to 3.23 Gy and 2.20 Gy under condition of 5 mm deep set-up error, respectively. Automated heart edge detection in cine MV image has been proposed [17]. If such technology is commercialized, adaptive radiotherapy could be applied to systematically monitor cardiac dose so that cardiac dose can be controlled below the constraint of each institution.

It is expected that set-up error can be controlled within about 4 mm by utilizing currently developed technology. In comparison with conventional laser-based set-up, surface guided radiotherapy using optical surface scanning system (OSS) can significantly reduce set-up errors, showing that 95% of fractions are within the clinical action level of ≤ 4 mm in any direction [18]. Patients with frequent set-up errors require more thorough management. Patients with uncertainty of initial treatment associated with inter-fractional variation should be carefully observed in the entire treatment period [19].

As DIBH requires holding the breath for more than 20 s and maintaining the same posture, reproducibility during radiotherapy is an important issue. A study estimating intra-fractional error using real time monitoring of OSS has presented that the mean motion during DIBH is small with < 1 mm translational and 1° rotational deviation [20]. In another study, set-up error during DIBH was measured using continuous portal imaging in 58 patients. The standard deviation of intra-fractional motion was 0.5 mm. However, large error exceeding 5 mm was occasionally presented in 12.1% of patients [21]. Cardiac motion affects cardiac dose. Distance variation from systolic to diastole was ≤ 4 mm for the LV and ≤ 3 mm for the heart and the LAD with a maximum dose of 5.2 Gy for the LV and a mean dose difference of 4.6 Gy for the LAD [22].

For lung cancer, lung dose constraint such as mean lung dose < 20 Gy, V20 Gy < 30%, V5 Gy < 65%, and absolute volume lung spared > 5 Gy, < 500 ml was recommended to protect radiation induced lung injury [23]. In a systemic review of recent reports regarding lung dose of breast radiotherapy, the average mean ipsilateral lung dose was 8.4 Gy for whole breast radiotherapy without breathing adaptation [24]. Therefore, the occurrence of lung toxicity is modest. Symptomatic pulmonary events of grade 2 developed in 2.7% of whole breast radiotherapy in actual modern practice [25]. The increase of mean lung dose was significantly correlated with lower lung volume and larger treatment volume [26]. In our study, the mean ipsilateral lung dose increased approximately 1 Gy per 2.5 mm deeper set-up, reaching 10 Gy provided that the action level was 5 mm deep for the heart.

Although excluded from the evaluation of this study, the dose distribution of CTV would be essential as much as OARs. The sub-fields of field-in-field technique are manually reconstructed taking account of the dose clouds in each tangential field and the broad deviation of dose distribution for CTV could be developed according to characteristics of breast contour and physician’s principle [27]. Therefore, the dose distribution of CTV related to the extent of caudal and deep set-up error could not show the uniform pattern on a case by case basis. For CTV or tumor bed, it is necessary that the set-up variation would be investigated in the different setting of tumor location (e.g. deep seated tumor, tumor on border of tangential field) and other direction of set-up error (e.g. shallow set-up) in further study.

This study considered the coverage of CTV as the most important factor when making a radiation plan without modifying the plan according to the proximity of the heart. Due to such principle, the original plan dose was somewhat high, especially for LAD. The DEGRO expert panel recommends cardiac dose constraints as mean heart dose < 2.5 Gy; mean LV dose < 3 Gy; V5 Gy of LV < 17%; V23 Gy of LV < 5%; mean LAD dose < 10 Gy; V30 Gy of LAD < 2%; and V40 Gy of LAD < 1% [28]]. In actual treatment, if a radiation field is tailored by weighting the location of tumor bed and heart toxicity, it will be possible to maintain a cardiac dose as low as reasonably achievable. In addition, since this study was not a comparative evaluation of the set-up of DIBH and FB in the same patient, there might be errors depending on body contour of the selected patient. Lastly, it is important to note that in actual treatment, uncertainty of set-up may complexly occur besides our deep and caudal set-up. However, the evaluation was performed on the premise of a deep and caudal set-up.

## Conclusions

Relatively modest set-up errors can meaningfully increase doses to the lung and heart. Under a deep set-up error within 5 mm, mean heart and ipsilateral lung doses increased up to 49.4% and 26.1% of original plan dose in DIBH, respectively. Compared to FB, DIBH can reduce the relative cardiac dose for the same extent of set-up errors in left breast cancer. It is necessary to keep in mind that radiation with a higher dose than the planned dose in actual radiation treatment could be irradiated. Thus, it is important to establish an action level for a set-up error suitable for treatment circumference of each institution.

## Data Availability

The data that support the findings of this study are available in Ansan Hospital, Korea University.

## Abbreviations

DIBH: Deep inspiration breath holding
FB: Free breathing
LV: Left ventricle
LAD: Left anterior descending coronary artery
CTV: Clinical target volume
CT: Computed tomography
OAR: Organs at risk
LSD: Least significant difference
OSS: Optical surface scanning system
